# β_2_-Adrenoceptor Activation Favor Acquisition of Tumorigenic Properties in Non-Tumorigenic MCF-10A Breast Epithelial Cells

**DOI:** 10.3390/cells13030262

**Published:** 2024-01-30

**Authors:** Dany Silva, Clara Quintas, Jorge Gonçalves, Paula Fresco

**Affiliations:** 1 Laboratory of Pharmacology, Department of Drug Sciences, Faculty of Pharmacy, University of Porto, 4050-313 Porto, Portugal; up201708266@edu.ff.up.pt (D.S.); claraquintas@ff.up.pt (C.Q.); pfresco@ff.up.pt (P.F.); 2 UCIBIO—Applied Molecular Biosciences Unit, Associate Laboratory i4HB, Institute for Health and Bioeconomy, University of Porto, 4050-313 Porto, Portugal

**Keywords:** breast cancer, adrenoceptors, carcinogenesis, cell death, adhesion, proliferation, migration

## Abstract

Noradrenaline and adrenaline, and their cognate receptors, are currently accepted to participate in cancer progression. They may also participate in cancer initiation, although their role in this phase is much less explored. The aim of this work was to study the influence of adrenergic stimulation in several processes related to breast cancer carcinogenesis, using several adrenergic agonists in the MCF-10A non-tumorigenic breast cells. Activation of the β-adrenoceptors promoted an epithelial phenotype in MCF-10A cells, revealed by an increased expression of the epithelial marker E-cadherin and a decrease in the mesenchymal markers, N-cadherin and vimentin. MCF-10A cell motility and migration were also impaired after the β-adrenoceptors activation. Concomitant with this effect, β-adrenoceptors decrease cell protrusions (lamellipodia and filopodia) while increasing cell adhesion. Activation of the β-adrenoceptors also decreases MCF-10A cell proliferation. When the MCF-10A cells were cultured under low attachment conditions, activation the of β- (likely β_2_) or of α_2_-adrenoceptors had protective effects against cell death, suggesting a pro-survival role of these adrenoceptors. Overall, our results showed that, in breast cells, adrenoceptor activation (mainly through β-adrenoceptors) may be a risk factor in breast cancer by inducing some cancer hallmarks, providing a mechanistic explanation for the increase in breast cancer incidences that may be associated with conditions that cause massive adrenergic stimulation, such as stress.

## 1. Introduction

Breast cancer remains a major health concern, globally. It is the most common and is the cause of the biggest number of cancer-related deaths in women [1]. To reduce its burden, understanding of the mechanisms which contribute to breast cancer appearance and development is fundamental.

Several studies have shown that noradrenaline and adrenaline, which are sympathetic transmitters, may exert a prominent role in promoting several types of cancers, namely breast cancer [2,3,4,5]. Effects of adrenaline and noradrenaline are mediated by G-protein coupled receptors, globally called adrenoceptors. Adrenoceptors are divided into three types and nine subtypes: α_1_ (α_1A_, α_1B_, α_1D_), α_2_ (α_2A_, α_2B_, α_2C_) or β (β_1_, β_2_, β_3_) and the effects on the breast are mainly mediated by β-adrenoceptors [6,7,8,9,10].

Noradrenaline is mainly released locally, upon stimulation of sympathetic nerve fibers, whereas adrenaline preferentially reaches the tissues through the blood circulation, after activation of the sympathetic–adrenal axis [7,8,11,12,13,14]. In the breast, noradrenaline and adrenaline control the development and branching of the mammary epithelial end buds and alter the lactation phase, influencing milk formation and its composition [7,8,10].

Under stress conditions, plasma levels of adrenaline and noradrenaline increases markedly, preparing the organism to a physiological pattern of “fight or flight” responses [15]. However, chronic stress is a cause of several diseases, being accepted to increase the incidence of cancer [16,17,18]. In mice, chronic exposure to stress has been shown to accelerate the onset of cancer/increased risk of mammary carcinoma [19,20,21], whereas women exposed to stressful life events have an higher risk of having breast cancer [22]. The adrenergic contribution to the stress-induced carcinogenesis is likely, since observational studies have shown that patients using β-adrenoceptor antagonists presented lower cancer incidence rates [23], whereas breast cancer patients receiving perioperative β-adrenoceptor antagonists presented lower cancer recurrence and less metastases [24]. Clinically, these effects may have an impact on the efficacy of chemotherapy, since administration of a β-adrenoceptor antagonist associated to trastuzumab plus chemotherapy achieved a significantly better progression-free survival in breast cancer patients comparatively to those treated only with trastuzumab plus chemotherapy [4].

Several mechanisms have been suggested to explain the adrenergic contribution for carcinogenesis. Adrenergic activation was shown to induce alterations in cells stemness [25] and on cancer cell capacity to migrate and invade [26,27,28,29]. The available evidence supports mainly the role of the adrenergic carcinogenesis in promoting progression or favoring initiation caused by other carcinogenetic stimuli [5]. The activation of β-adrenoceptors with a non-selective β-adrenoceptor agonist was reported to increase incidences of chemically induced breast cancer (using methyl nitrosourea; NMU) in mice [5], whereas rats treated with propranolol concomitantly to NMU revealed lower incidence rates of mammary carcinoma [30]. Whether adrenergic stimulation can, per se, promote the acquisition of carcinogenic properties of non-carcinogenic cells (cancer initiation) remains to be elucidated. 

The aim of the present study was to investigate the influence of adrenoceptor activation on several cellular processes known to be important in the initiation of breast cancer, by carrying out an exploratory study to investigate whether adrenergic activation can promote the acquisition of tumorigenic properties in non-tumorigenic breast cells. For this purpose, a spontaneous immortalized human non-tumorigenic cell line (MCF-10A cells), widely used as a non-tumorigenic cell line, was used and the adrenergic influence on the epithelial/mesenchymal phenotype, cell migration and motility, adhesion, proliferation, and resistance to cell death was investigated. This research will be focused mainly in the α_2_- and the β-adrenoceptor subtypes, since the available evidence indicates that these receptor subtypes are mainly involved in carcinogenic responses [31].

The epithelial-to-mesenchymal transition (EMT) and the reverse process, mesenchymal–epithelial transition (MET), are processes that have been shown to be important during carcinogenesis cascade [32,33]. Alterations in EMT/MET are considered cancer hallmarks and play a role in cancer initiation and in breast cancers invasion [34,35,36,37]. Cell–matrix adhesion is also found to be altered during carcinogenesis, affecting several cellular processes related to cancer [38,39]. Cell–matrix adhesion may affect cell migration, as this process involves a highly dynamic coordination between cell protrusions formation and cell–matrix adhesion, and cell survival under conditions of insufficient or inappropriate cell–matrix interactions [38,39,40]. Non-tumorigenic cells cannot survive under loss/inappropriate matrix adhesion and die by activating cell death mechanisms [41,42]. However, under certain conditions, cells can acquire mechanisms to support cell survival after the loss of attachment conditions [40,43,44,45]. The acquisition of such mechanisms has pathological relevance, since it has been linked to the formation of ductal carcinomas in situ, which are premalignant lesions of the breast [40,43,44,45]. Tumorigenic cells also have a higher cell proliferation than non-tumorigenic cells due to a sustaining proliferative signaling [46]. The present study showed that adrenoceptor (mainly β_2_-adrenoceptors) activation in MCF-10A cells can regulate some cancer hallmarks, providing a mechanistic explanation for the increase in breast cancer incidence that may be caused by the adrenergic stimulation known to occur during stress.

## 2. Materials and Methods

### 2.1. Chemicals

The following compounds were purchased from Sigma-Aldrich (Chemicalnor, Valongo, Portugal). (-)-Adrenaline, (-)-isoprenaline, salbutamol, UK 14,304 [5-bromo-N-(4,5-dihydro-1H-imidazol-2-yl)quinoxalin-6-amine], (±)-propranolol, Dulbecco’s Modified Eagle’s Medium/F-12, epidermal growth factor, human insulin, hydrocortisone and penicillin/streptomycin. L-glutamine was purchased from Gibco (Biotecnómica, São Mamede de Infesta, Portugal). Biolegend (Lusopalex, Lisboa, Portugal) provided Flash Phalloidin™ Green 488 while foetal bovine serum was obtained from Sigma-Aldrich (Biotecnómica). Seahorse low melting agarose was from Serva (Fisher Scientific, Madrid, Spain). PrestoBlue^TM^ reagent and 0.25% Trypsin/0.025% EDTA solution were purchased from Invitrogen and Gibco, respectively (Alfagene, Carcavelos, Portugal).

### 2.2. Cells, Culture Conditions and Treatments

The MCF-10A breast epithelial cells (ATCC cat#CRL-10317, RRID:CVCL_0598; LGC Standards, Barcelona, Spain) were cultured in a DMEM/F12 culture medium and supplemented with the following compounds: 20 ng/mL epidermal growth factor; 2 mM of stable L-glutamine, 3.5 μg/mL human insulin, 0.5 μg/mL hydrocortisone, 10% heat-inactivated FBS, and 1% of a solution composed of penicillin/streptomycin. Cells were maintained in a humidified environment comprising 95% air and 5% CO_2_, at a temperature of 37 °C. Cell subculturing was performed twice a week using trypsinization (0.25% trypsin/0.025% EDTA), ensuring that cells were maintained below a 90% confluence. Regular mycoplasma contamination tests were conducted on the cells.

Prior to each experimental assay, MCF-10A cells underwent trypsinization and were centrifuged at 457× *g* for five minutes at 20 °C. Cells were seeded onto 96- or 6-well plates (TPP, Biotecnómica) at various densities, according to the experimental assay. Depending on the experimental objectives, cells were exposed to isoprenaline (0.1–10 μM), adrenaline (0.1–10 μM), salbutamol (0.1–10 μM), UK 14,304 (0.1–10 μM), or propranolol (10 μM), either individually or in combination, and then incubated for up to 72 h. Control experiments included cells treated with the vehicle alone (0.1% DMSO) in each assay.

### 2.3. Cell Adhesion Assays

To probe cell adhesion, two different experimental approaches were performed, depending on the experimental time point that was on study: the wash assay technique was used for the 1 h incubation time point and the trypsin detachment assay was used for the 24 and 72 h incubation periods.

The wash assay technique was performed as described before [47]. Briefly, suspended MCF-10A cells were treated with different adrenergic agonists/antagonists (Section 2.2) in parallel with the respective control (vehicle), at a density of 6.0 × 10^3^ cells/well. Cells were then seeded in 96-well plates and allowed to incubate for 1 h. At the end of this incubation period, cells were gently washed with a warmed culture medium. Adherent cells were incubated with PrestoBlue^TM^ reagent, for 1.5 h at 37 °C, and the fluorescence in each well was measured using a microplate reader (Synergy HT, BioTek Instruments Inc., Winooski, VT, USA). A linear curve was achieved for the plotting of cell density and the PrestoBlue^TM^ reagent fluorescence signal (r^2^ = 0.99). All conditions were performed in triplicates. Results were expressed as a percentage of the respective control.

Cell adhesion assays using trypsin were performed as described [48], with minor modifications. The MCF-10A cells were seeded in 96-well plates, at an initial density of 4.0 × 10^3^ cells/well. After a 24 h incubation, the culture medium was carefully removed and different adrenergic agonists/antagonists were added into the fresh culture medium (Section 2.2) in parallel with the respective controls (vehicles), for 24 h or 72 h. At the end of the respective incubation period, cells were washed with warm PBS and incubated with trypsin solutions of different dilutions (1:5.5 at 24 h and 1:5 at 72 h, in PBS) for 10 min. At the end of this incubation period, cells were gently washed with a warm culture medium. Cells that remained adherent to the plate were incubated at 37 °C with PrestoBlue^TM^ reagent for 1.5 h, and fluorescence was measured using an automated microplate reader (Synergy HT, BioTek Instruments Inc.). To normalize for alterations in cell number induced by distinct treatments, wells where cells were not exposed to trypsin were included in the experiment and carried out in parallel. All conditions were performed in triplicates. Results were expressed as a percentage of the respective control.

### 2.4. Cell Proliferation Assay

Cell proliferation was performed using an automated label-free cell counting method named High Contrast Brightfield (HCB) counting, as previously described [49]. For these experiments, MCF-10A cells were seeded in 96-well plates, at an initial density of 4.0 × 10^3^ cells/well. After 24 h incubation, the culture medium was removed, and cells were treated with different concentrations of adrenergic agonists/antagonists (see Section 2.2) in parallel with the respective control (vehicle) for up to 72 h. During this incubation period, the number of cells in each well were monitored at different time points (0, 24, 48, and 72 h) by capturing HCB images of the center of each well, using an automated inverted microscope (Lionheart FX microscope, BioTek Instruments, Inc.). Gen5 v3.04 image analysis software (BioTek Instruments Inc.) was used to stitch and pre-process the acquired images, and to perform cell masking thresholds to identify each cell. Results for the number of cells obtained at each time point were normalized to the initial cell number (time point 0 h). In each plate, all conditions were performed, at least, in triplicates. Results were then expressed as percentage of the respective control.

### 2.5. Random Motility Assay

The MCF-10A cells were seeded in 96-well plates, at an initial density of 4.0 × 10^3^ cells/well. After 24 h incubation, the culture medium was carefully removed, and cells were treated with different concentrations of adrenergic agonists and antagonists (Section 2.2) in parallel with the respective control (vehicle used for the preparation of drugs, at the same final concentration) for 24 h. Cells were then transferred to an automated inverted microscope (Lionheart FX, BioTek Instruments Inc.) with a humidified and controlled atmosphere (37 °C, 5% CO_2_). HCB images of cells were captured every hour for a 24 h period. Gen5 v3.04 image analysis software (BioTek Instruments Inc.) was then used to stitch and pre-process the acquired Lionheart FX images. Quantification of individual cell motility was then performed using ImageJ v.1.53 (using the Manual Tracking plugin). In each individual experiment, all conditions were performed, at least, as duplicates.

### 2.6. Wound Healing Assay

Directed/collective migration was monitored using the wound healing assay, as previously described [50], with minor modifications. Briefly, the MCF-10A cells were seeded in 96-well plates, at an initial density of 2.0 × 10^4^ cells/well. After 24 h of incubation, scratches were carefully made across the confluent cell monolayers using a 20 μL sterile pipette tip. Cells were then exposed to adrenergic agonists/antagonists, at different concentrations (Section 2.2) in parallel with the respective control (solvent/vehicle), and transferred to an automated inverted microscope (Lionheart FX, BioTek Instruments Inc.) with a humidified and controlled atmosphere (37 °C, 5% CO_2_). Brightfield images of the scratches were captured every hour, for a 24 h time period. Quantification of the scratch areas were performed using ImageJ v.1.53 (Wound Healing Size Tool plugin). Scratch segmentations were visually checked and, when necessary, manual editing of the scratch analysis was performed. All conditions were performed as duplicates, at least. Results of the scratch areas were normalized by the initial scratch area for each well.

### 2.7. F-Actin Staining, Cell Morphometric Analysis, and Cell Protrusions Quantification

The MCF-10A cells were seeded in 96-well plates, at an initial density of 2.0 × 10^3^ cells/well. After 24 h incubation, the culture medium was carefully removed, and cells were treated with adrenergic agonists/antagonists at different concentrations (Section 2.2), in parallel with the respective control (vehicle) for either 24 h or 72 h.

Visualization of F-actin was performed using fluorescent phalloidin (Flash Phalloidin™ Green 488), according to the manufacturer’s protocol (Biolegend).

Briefly, the cells were fixed with 4% paraformaldehyde for 10 min, washed two times with PBS, and permeabilized with a solution containing 0.5% Triton X-100 in PBS for 10 min. Nonspecific binding sites were blocked by incubating cells with a solution containing 5% fetal bovine serum in PBS for 30 min. F-actin was stained by the incubating cells with Flash Phalloidin™ Green 488 (1:100 in PBS), for 20 min, protected from light. Cell nuclei were stained with Hoechst 33342 (concentration in the well: 5 μg/mL). Images were captured using the Lionheart FX inverted automated microscope (BioTek Instruments Inc.). Gen5 v3.04 image analysis software (BioTek Instruments Inc.) was used to stitch and pre-process the acquired images. An assessment of cell morphometry was performed by analyzing the F-actin-stained images. Quantification of the nuclei and cell area was performed using the Gen5 v3.04 image analysis software. For cell protrusions analysis, the presence or absence of lamellipodium and the number of filopodia *per* cell were manually counted in at least 50 cells *per* each condition. Results were then expressed as a percentage of the respective control.

### 2.8. mRrNA Extraction and RT-PCR

MCF-10A cells were seeded in 6-well plates at an initial density of 1.1 × 10^5^ cells/well. After 24 h of incubation, the culture medium was carefully removed and cells were treated with isoprenaline (10 μM) or propranolol (10 μM), alone or in combination, in parallel with the respective control (medium) for 24 h. At this time point, RNA extraction was carried out using the RNeasy Mini Kit from QIAGEN (Werfen, Carnaxide, Portugal), following the manufacturer’s instructions. RNA purity and concentration was confirmed by spectrophotometry using a microplate reader (Synergy HT, BioTek Instruments Inc). In the reverse-transcriptase reactions, 1000 ng of extracted RNA served as a template, utilizing the Xpert cDNA Synthesis Mastermix kit from Grisp (Biotecnómica). The following primer sequences were used: 

E-cadherin–F: CAATGCCGCCATCGCTTAC; R: ATGACTCCTGTGTTCCTGTTAATG; N-cadherin–F: ATGGTGTATGCCGTGAGA; R: CAACTTCTGCTGACTCCTTC; 

Vimentin–F: ACCAAGACCTGCTCAATG; R: CAACCAGAGGGAGTGAATC.

NCBI BLAST analysis was employed to validate primer specificity. Subsequent to RT-PCR, further confirmation of primer specificity was ensured by assessing the dissociation curve with only one single peak, with an observed Tm (primer melting temperature) aligned with the amplicon length. The relative efficiency and quality of primers were also checked by using cDNA standard dilutions. No template cDNA was used as negative control in the qPCR experiment. qPCR amplifications were performed and analyzed as previously described by Amaro et al. [51]. β-actin and GAPDH were used as reference genes for normalization. Raw Ct values of the three studied genes and also the two reference genes for all treatment groups are shown in Supplementary Figure S1.

### 2.9. Resistance to Cell Death under Low Attachment Conditions

The resistance to cell death under low attachment conditions was performed as previously described [52], with minor modifications. MCF-10A cells were seeded in 96-well plates, pre-coated with 1% low melting agarose, at an initial density of 1.0 × 10^4^ cells/well. The cells were immediately treated with adrenergic agonists/antagonists (Section 2.2) in parallel with the respective control (cells with vehicle) and allowed to incubate for up to 72 h. At the end of each incubation period, cells were incubated with PrestoBlue^TM^ reagent for 1 h at 37 °C, and fluorescence was measured using an automated microplate reader (Synergy HT, BioTek Instruments Inc.). In a set of experiments, cells were incubated with Hoechst 33342 (5 μg/mL) and Ethidium bromide (1 μg/mL) for 15 min at 37 °C. Fluorescence images of cell aggregates and dead cells were acquired using the Lionheart FX microscope and analyzed with the Gen5 v3.04 software (BioTek Instruments Inc.). All conditions were performed in triplicates. Results were expressed as a percentage of the respective control.

### 2.10. Statistical Analysis

Graph and statistical analysis were carried out using GraphPad Prism 8 software. The present study was an exploratory study, and therefore, the *p*-values should be interpretated as descriptive. The differences between controls and treatments were compared using an one-way ANOVA with repeated measures, followed by the post hoc multi-comparisons Dunnett’s test. Otherwise stated, the differences between controls and treatments were also calculated using a two-way ANOVA followed by the post hoc multi-comparisons Dunnett’s test or the Šídák test. Differences between treatments were evaluated using Student’s *t*-test, or a two-way ANOVA followed by the post hoc multi-comparisons Šídák test. *p* < 0.05 values were indicative of statistically significant differences.

## 3. Results

### 3.1. β2-Adrenoceptor Activation Alters in MCF-10A Cell Morphology

**Figure 1:**
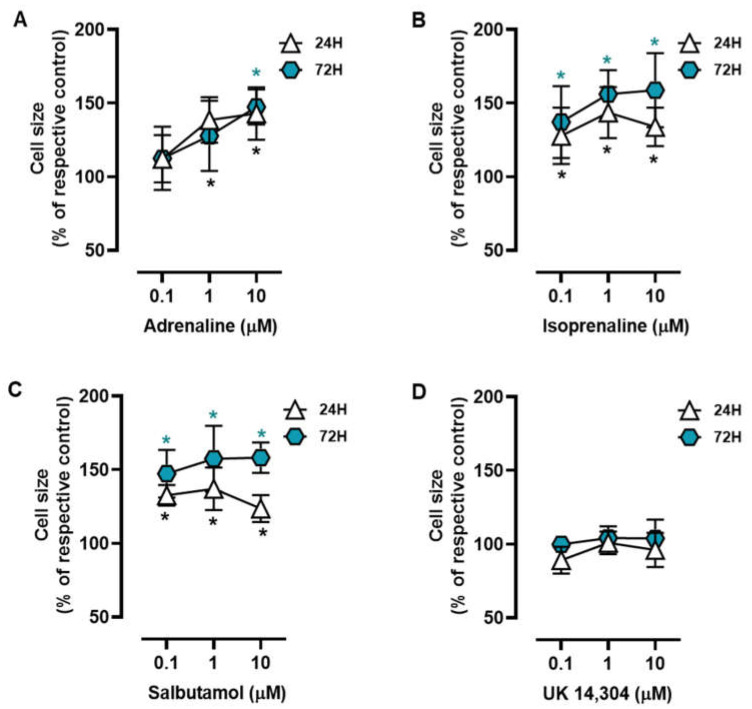
Influence of β- and α_2_-adrenoceptor agonists on MCF-10A cell size. Cells were treated with either the non-selective adrenoceptor agonist adrenaline (**A**), the non-selective β-adrenoceptor agonist isoprenaline (**B**), the β_2_-adrenoceptor agonist salbutamol (**C**) or with the α_2_-adrenoceptor agonist UK 14,304 (**D**) for 24 (triangle) or 72 h (diamonds). Results are expressed as percentage of control (solvent) and are presented as mean ± SD from five independent experiments. Significantly different from solvent: * *p* < 0.05; one-way ANOVA with repeated measures, post hoc multi-comparisons Dunnett’s test.

MCF-10A cells were treated with different adrenoceptor agonists, with an affinity for either β- or α_2_-adrenoceptors, the two main adrenoceptor types were shown to be functional in breast cells [10,31,51,53]. Morphometric analysis of the MCF-10A cells treated with the non-selective endogenous adrenoceptor agonist adrenaline (0.1–10 μM) or the β-adrenoceptor agonist isoprenaline (0.1–10 μM) revealed that these agonists caused an increase in the MCF-10A cell area (Figure 1A and Figure 1B, respectively). Salbutamol (0.1–10 μM), a selective β_2_-adrenergic agonist, also had a similar effect to that exerted by isoprenaline or adrenaline on the MCF-10A cell area (Figure 1C). The selective α_2_-adrenoceptor agonist UK 14,304 (0.1–10 μM) did not cause any effect in the MCF-10A cell area (Figure 1D). Further analysis of the calculated ratios nuclei area:cell area did not reveal any change in cells treated with isoprenaline, adrenaline, or salbutamol (Supplementary Figure S2), which was taken as an indication that β-adrenoceptors agonists were causing a parallel increase in nuclei (likely a decrease in chromatin condensation) and cytoplasmic area.

### 3.2. β2-Adrenoceptor Activation Increases MCF-10A Cell-to-Matrix Adhesion

**Figure 2:**
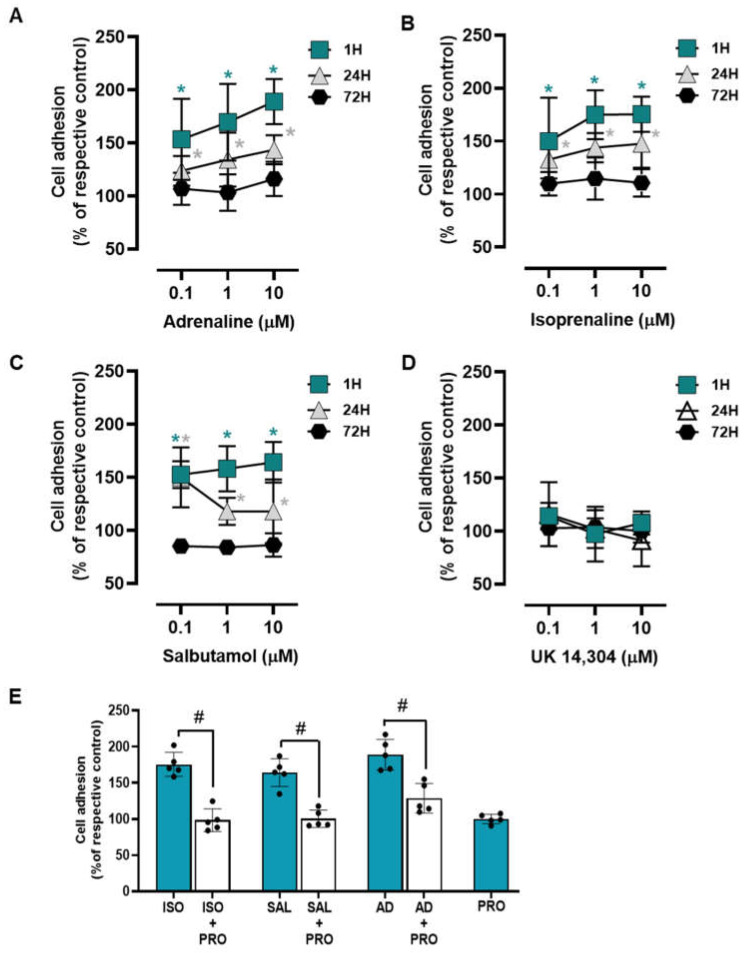
Influence of β- and α_2_- adrenoceptor agonists on MCF-10A cell adhesion. (**A**–**D**) Results for MCF-10A cell adhesion after treatment with either the non-selective adrenoceptor agonist adrenaline (**A**), the non-selective β-adrenoceptor agonist isoprenaline (**B**), the β_2_-adrenoceptor agonist salbutamol (**C**) or with the α_2_-adrenoceptor agonist UK 14,304 (**D**) for 1 (square), 24 (triangle) or 72 h (diamonds). Cell adhesion after 1 h incubation was measured using the wash assay technique. Cell adhesion after 24 and 72 h incubation was measured using trypsin as the detaching agent. (**E**) Influence of the β-adrenoceptor antagonist propranolol (PRO; 10 μM), alone or in the presence of isoprenaline (ISO; 10 μM), salbutamol (SAL; 10 μM) or adrenaline (AD; 10 μM) after 1 h incubation using the wash assay technique. Results are expressed as percentage of the respective control and are presented as mean ± SD from five independent experiments. Significantly different from solvent: * *p* < 0.05; one-way ANOVA with repeated measures, post hoc multi-comparisons Dunnett’s test. Significantly different from the agonist alone: ^#^ *p* < 0.05, Student’s *t*-test.

To examine whether adrenoceptor activation affects the adhesion of the MCF-10A to culture matrix, cells were incubated with adrenoceptor agonists for different incubation periods (1 h; 24 h; 72 h). Both adrenaline and isoprenaline (tested at 0.1–10 μM) increased cell to a matrix adhesion both at 1 h and 24 h incubation periods (Figure 2A and Figure 2B, respectively). At longer incubations periods (72 h) both agonists lost this ability and showed no effect on MCF-10A cell-to-matrix adhesion. The β_2_-adrenergic agonist salbutamol (0.1–10 μM) revealed a similar effect to those exerted by isoprenaline or adrenaline on MCF-10A cell adhesion (Figure 2C). The α_2_-adrenoceptor agonist UK 14,304 (0.1–10 μM) did not cause any effect in MCF-10A cell adhesion at all incubation periods tested (Figure 2D), suggesting that α_2_-adrenoceptors do not play a role in MCF-10A cell adhesion. These results suggest that the adrenoceptor mediated effect on cell adhesion may be ascribed to the activation of β-adrenoceptors, mainly of the β_2_-subtype.

A different set of experiments were carried out using the adrenoceptor agonists (isoprenaline, salbutamol and adrenaline; tested at 10 μM) and their combinations with the β-adrenoceptor antagonist propranolol (10 μM). Experiments were conducted after a 1 h incubation period, as this was the experimental condition where the highest increase in cell adhesion was observed in the previous experiments. This set of experiments showed that propranolol was able to completely abrogate the agonists’ effects (Figure 2E). These findings reinforce the hypothesis of a major role for β-adrenoceptor activation in the MCF-10A cells’ increased adhesion to matrix.

### 3.3. β2-Adrenoceptor Activation Decreases MCF-10A Cell Proliferation

**Figure 3:**
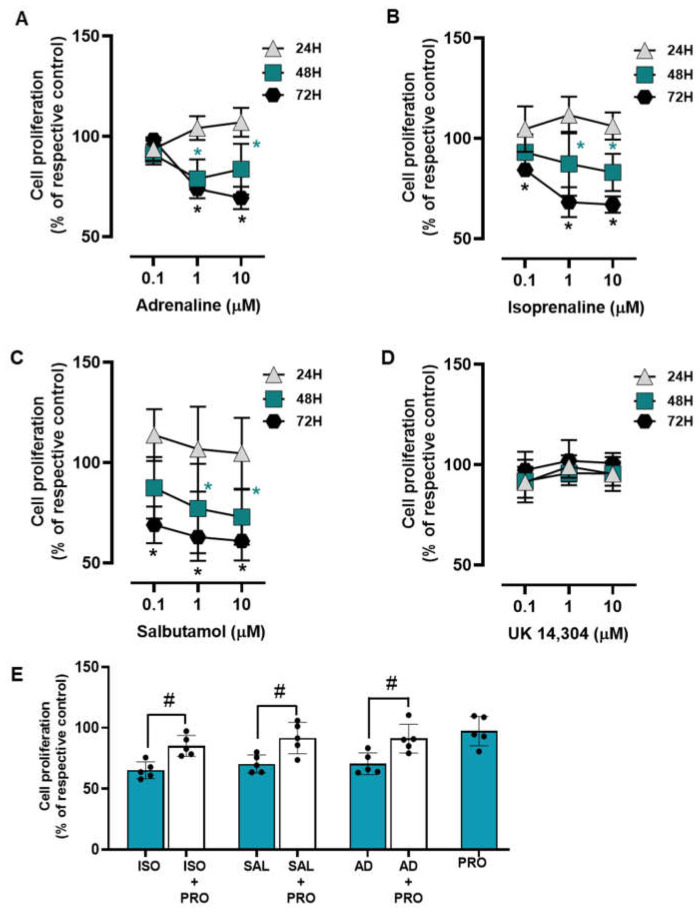
Influence of β- and α_2_-adrenoceptor agonists on MCF-10A cell proliferation. (**A**–**D**) Results for MCF-10A cell proliferation after treatment with either the non-selective adrenoceptor agonist adrenaline (**A**), the non-selective β-adrenoceptor agonist isoprenaline (**B**), the β_2_-adrenoceptor agonist salbutamol (**C**) or the α_2_-adrenoceptor agonist UK 14,304 (**D**) for 24 (triangle), 48 (square) or 72 h (diamonds). (**E**) Influence of the β-adrenoceptor antagonist propranolol (PRO; 10 μM, alone or in the presence of isoprenaline (ISO; 10 μM), salbutamol (SAL; 10 μM) or adrenaline (AD; 10 μM) on MCF-10A cell proliferation after incubation for 72 h. Results are expressed as percentage of control (solvent) and are presented as mean ± SD from four to five independent experiments. Significantly different from solvent: * *p* < 0.05; one-way ANOVA with repeated measures, post hoc multi-comparisons Dunnett’s test. Significantly different from the agonist alone: ^#^ *p* < 0.05, Student’s *t*-test.

The influence of the adrenoceptor agonists was tested on MCF-10A cell proliferation after incubation periods of 24, 48, and 72 h. All adrenoceptors’ agonists with an affinity for β-adrenoceptors [adrenaline, isoprenaline and salbutamol, tested at 0.1–10 μM] decreased MCF-10A cell proliferation; the α_2_-adrenoceptor agonist UK 14,304 did not cause any effect on MCF-10A cell proliferation (Figure 3). These results suggest that adrenoceptor activation decreases cell proliferation in MCF-10A cells, involving activation of β- but not of α_2_-adrenoceptors.

The involvement of β-adrenoceptors in the decrease of cell proliferation caused by adrenoceptor agonists was further investigated by testing their effects on the absence or in the presence of propranolol (10 μM). As shown in Figure 3E, propranolol alone did not alter cell proliferation, but prevented the decrease in cell viability caused by adrenaline, isoprenaline, and salbutamol (all tested at 10 μM).

### 3.4. β2-Adrenoceptor Activation Decreases MCF-10A Cell Motility

**Figure 4:**
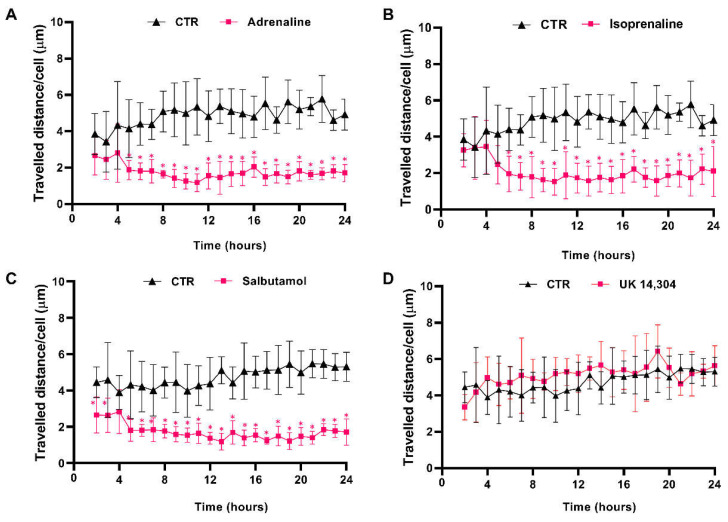
Influence of β- and α_2_-adrenoceptor agonists on MCF-10A cell motility. Cells were treated with either the non-selective adrenoceptor agonist adrenaline (**A**); 10 μM), the non-selective β-adrenoceptor agonist isoprenaline (**B**); 10 μM), the β_2_-adrenoceptor agonist salbutamol (**C**); 10 μM) or with the α_2_-adrenoceptor agonist UK 14,304 (**D**); 10 μM) for 24 h. Results presented as mean ± SD from four to five independent experiments. Significantly different from respective solvent: * *p* < 0.05; two-way ANOVA, followed by the post hoc multi-comparisons Šídák test. CTR—control.

The effects of β- and α_2_-adrenoceptor agonists on MCF-10A cells motility were first evaluated using timelapse microscopy experiments to track MCF-10A random cell movement. These cells have been described, previously, as presenting high random motility [54]. This finding was confirmed by our observations (see Supplementary Figure S3). Moreover, all adrenergic agonists, with an affinity for β-adrenoceptors (adrenaline, isoprenaline, and salbutamol, tested at 10 μM) inhibited MCF-10A cell motility, whereas the α_2_-adrenergic agonist UK 14,304 (10 μM) had no effect on MCF-10A random cell motility (Figure 4). Consistently, the average movement velocity of MCF-10A cells during the 24 h incubation period decreased about 2× with the treatment with 10 μM isoprenaline (control cells: 4.9 μm/h vs. cells treated with isoprenaline: 2.0 μm/h), whereas in MCF-10A cells treated with 10 μM salbutamol or 10 μM adrenaline, this parameter decreased approximately 3× (control: 4.5 μm/h vs. cells treated with salbutamol: 1.7 μm/h; control cells: 4.9 μm/h vs. cells treated with adrenaline: 1.8 μm/h).

### 3.5. β2-Adrenoceptor Activation Decreases MCF-10A Cell Migration

**Figure 5:**
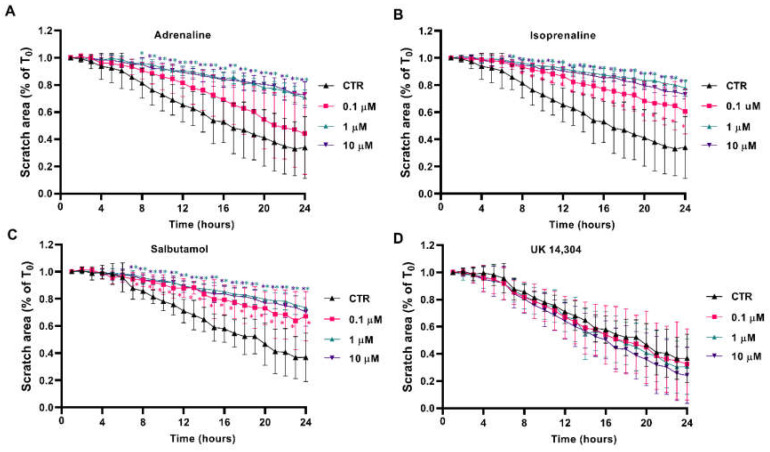
Influence of β- and α_2_-adrenoceptor agonists on MCF-10A cell migration, using the scratch assay. MCF-10A cells, grown until confluence, were scratched using a 20 μL tip and subsequently treated with either the non-selective adrenoceptor agonist adrenaline (**A**), the non-selective β-adrenoceptor agonist isoprenaline (**B**), the β_2_-adrenoceptor agonist salbutamol (**C**) or with the α_2_-adrenoceptor agonist UK 14,304 (**D**) for 24 h. Results are expressed as a percentage of the initial scratch area (T_0_) and presented as mean ± SD from four independent experiments. * *p* < 0.05; two-way ANOVA, followed by the post hoc multi-comparisons Dunnett’s test. CTR—control.

Scratch/wound healing assays were performed to evaluate the adrenergic influence on collective MCF-10A cell migration, which measures the contribution of directed migration induced by the scratch conditions [55]. In our experimental conditions, MCF-10A cells treated with adrenergic agonists with affinity for β-adrenoceptors (adrenaline, isoprenaline and salbutamol, tested at 0.1–10 μM) had a reduced ability to migrate into the scratch area (Figure 5A, Figure 5B, Figure 5C, respectively). By opposition, incubation of the MCF-10A cells with the α_2_-adrenoceptor agonist UK 14,304 did not influence MCF-10A cells migration (Figure 5D).

The involvement of β-adrenoceptors on the decrease in cell migration caused by adrenoceptor agonists was further investigated by testing their effects on the absence or in the presence of propranolol (10 μM). As shown in Supplementary Figure S4, propranolol alone did not alter cell migration but prevented the decrease in cell migration caused by adrenaline, isoprenaline and salbutamol (all tested at 10 μM). These findings suggest a major role for β-adrenoceptor activation in decreasing MCF-10A cell motility.

### 3.6. β2-Adrenoceptor Activation Decreases MCF-10A Cell Protrusions

**Figure 6:**
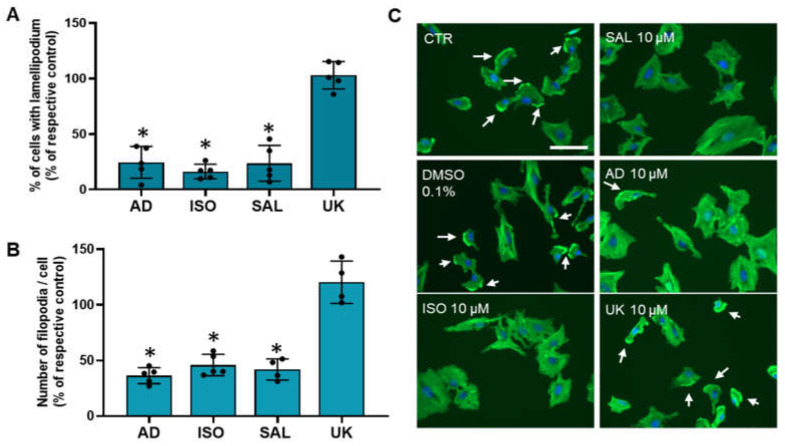
Influence of β- and α_2_-adrenoceptor activation on MCF-10A lamellipodia (**A**) or filopodia cell protrusions (**B**). Cells were treated with either the non-selective adrenoceptor agonist adrenaline (AD; 10 μM), the non-selective β-adrenoceptor agonist isoprenaline (ISO; 10 μM), the β_2_-adrenoceptor agonist salbutamol (SAL; 10 μM) or with the α_2_-adrenoceptor agonist UK 14,304 (UK; 10 μM) for 24 h. Results are expressed as percentage of control (solvent) and are presented as mean ± SD from four to five independent experiments. * *p* < 0.05; Student’s *t*-test (**C**) Representative images of F-actin labeling (green fluorescence) in the absence or presence of isoprenaline (ISO), salbutamol (SAL), adrenaline (AD) or UK 14,304 (UK). Nuclei were labeled with Hoechst 33342 (blue fluorescence). Scale bar: 100 μM. White arrows are indicative of the presence of lamellipodium.

Changes in cell motility/migration could arise, among other factors, from alter cell protrusions dynamics [54]. Therefore, it was also investigated whether treatments with adrenergic agonists could affect MCF-10A cellular protrusions (lamellipodia and filopodia). Immunofluorescence microscopy experiments using Flash Phalloidin^TM^, conjugated with a green fluorophore, were carried out. Lamellipodium were identified, by immunofluorescence as intense 2–3 μm green bands of F-actin near the cell periphery at the leading edge of all mobile cells and have been considered a major driver of cell motility [54,56]. Approximately 40 ± 7% of untreated MCF-10A cells presented lamellipodia. As shown in Figure 6A, the fraction of cells presenting lamellipodia drastically decreased after treatment with agonists with affinity for β-adrenoceptors (adrenaline, isoprenaline and salbutamol, all tested at 10 μM). In contrast, the α_2_-adrenoceptors agonist UK 14,304 (10 μM) did not alter the lamellipodia number (Figure 6A). Filopodia, which are spike-like actin protrusions, function as sensors of the local environment and have mechanical roles [57]. The number of these structures per cell were also reduced after treatment with β-adrenoceptor agonists, but not after treatment with the α_2_-adrenoceptor agonist UK 14,304 (see Figure 6B). Figure 6C shows representative images of the MCF-10A cells treated with β-adrenoceptors agonists (adrenaline, isoprenaline and salbutamol, all tested at 10 μM) showing a decrease in lamellipodia cell protrusions (white arrows).

### 3.7. β-Adrenoceptor Activation Increases MET Markers in MCF-10A Non-Tumorigenic Cells

**Figure 7:**
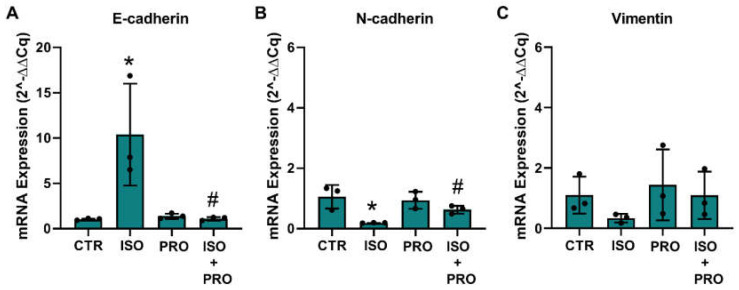
Influence of β-adrenoceptor activation on mRNA levels of (**A**) E-cadherin, (**B**) N-cadherin and (**C**) vimentin markers in MCF-10A cells determined by RT-PCR. Cells were treated for 24 h with the non-selective β-adrenoceptor agonist isoprenaline (ISO; 10 μM), or with the β-adrenoceptor antagonist propranolol (PRO; 10 μM), alone or in combination. Results were normalized to β-actin mRNA levels. Similar results were obtained when mRNA levels were normalized to that of GAPDH. Values were expressed as percentage of control (solvent/vehicle) and are presented as mean ± SD from three independent experiments. Significantly different from solvent: * *p* < 0.05; Significantly different from the agonist alone: ^#^ *p* < 0.05, Student’s *t*-test. CTR—control.

Epithelial-to-mesenchymal transition (EMT) and the reverse process, the mesenchymal–epithelial markers (MET), are processes that regulate migration and protrusions dynamics [58,59]. To investigate whether β-adrenoceptor activation could alter MCF-10A markers related to the epithelial or mesenchymal phenotype, cells were treated with the β-adrenoceptor agonist isoprenaline (10 μM), and mRNA levels of the epithelial marker E-cadherin and of the mesenchymal markers, N-cadherin and vimentin, were analyzed. GADPH and β-actin were used as reference genes. The effect of the drugs was compared using the respective mean ± SD of the raw Ct values and after normalization by each of the reference genes. Isoprenaline (10 μM) decreased the expression of β-actin (15.19 ± 0.65 vs 18.29 ± 0.07; control vs isoprenaline) and GADPH (16.86 ± 0.41 vs 20.09 ± 0.17) by about three units. Without normalization, isoprenaline further reduced the expression of the mesenchymal target genes N-cadherin (24.91 ± 1.01 vs 30.60 ± 0.23) and vimentin (17.12 ± 0.97 vs 22.03 ± 0.89), whereas the expression of E-cadherin did not change (29.13 ± 0.38 vs 29.11 ± 0.56). Propranolol (10 μM) alone did not alter the expression of β-actin (raw Ct value 15.19 ± 0.65 vs 15.33 ± 0.17; control vs propranolol) and GADPH (raw Ct value 16.86 ± 0.41 vs 17.13 ± 0.36). Normalization of the target gene expression by GADPH showed an increase in the expression of E-cadherin (Figure 7A), whereas the expression of N-cadherin and vimentin decreased, these effects being prevented by propranolol (Figure 7B,C); normalization of the target gene expression by β-actin is shown in Supplementary Figure S5).

### 3.8. β2-Adrenoceptor Activation Confers Protection against Cell Death in MCF-10A Cells under Low Attachment Conditions

**Figure 8:**
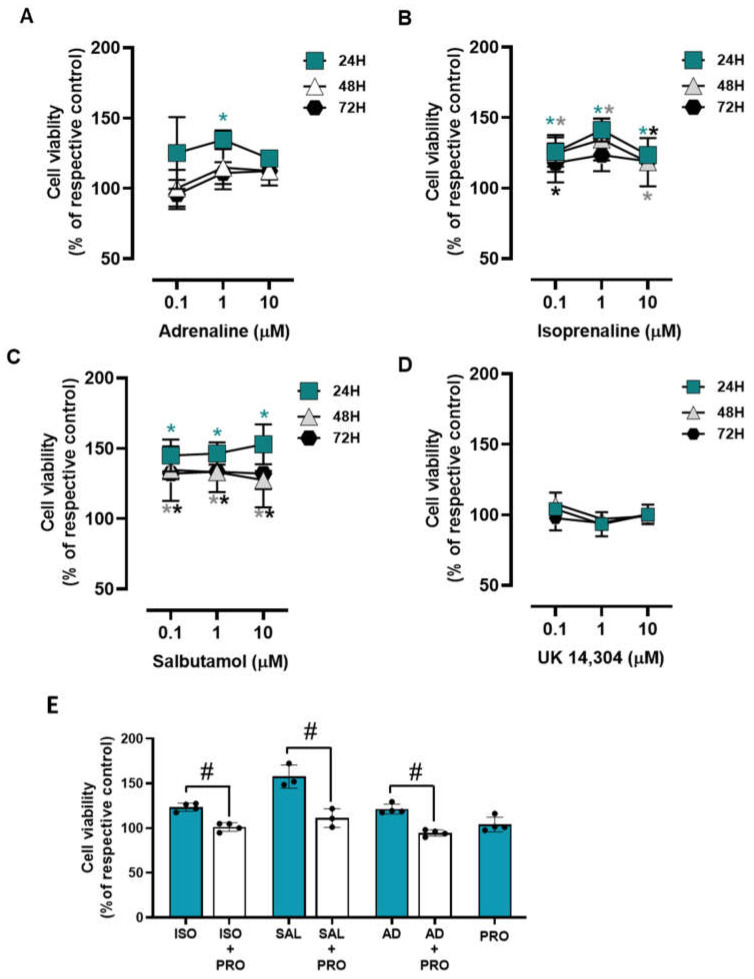
Influence of β- and α_2_-adrenoceptor agonists on MCF-10A cell survival under low attachment conditions. (**A**–**D**) Results for MCF-10A cell survival under low attachment conditions after treatment with either the non-selective adrenoceptor agonist adrenaline (**A**), the non-selective β-adrenoceptor agonist isoprenaline (**B**), the β_2_-adrenoceptor agonist salbutamol (**C**) or with the α_2_-adrenoceptor agonist UK 14,304 (**D**) for 24 (square), 48 (triangle) or 72 h (diamonds). (**E**) Influence of the β-adrenoceptor antagonist propranolol (PRO; 10 μM), alone or in combination with isoprenaline (ISO; 10 μM), salbutamol (SAL; 10 μM) or adrenaline (AD; 10 μM) on MCF-10A cell survival under low attachment conditions after 24 h of incubation. Results are expressed as percentage of control (solvent) and are presented as mean ± SD from four independent experiments. Significantly different from solvent: * *p* < 0.05; one-way ANOVA with repeated measures, post hoc multi-comparisons Dunnett’s test. Significantly different from the agonist alone: ^#^ *p* < 0.05, Student’s *t*-test; n.s.—non-significantly different from solvent.

To investigate whether adrenoceptor activation could affect cell survival under low attachment conditions, MCF-10A cells were plated in 1% agarose pre-coated well plates to simulate cell death and immediately treated with different adrenoceptor agonists. The MCF-10A cells treated with adrenergic agonists with an affinity for β-adrenoceptors (adrenaline, isoprenaline, and salbutamol, tested at 0.1–10 μM) showed an increase in cell viability under these conditions (Figure 8A, Figure 8B, Figure 8C, respectively), whereas the α_2_-adrenoceptor agonist UK 14,304 (0.1–10 μM) did not influence MCF-10A cell viability (Figure 8D).

The involvement of β-adrenoceptors on the increase in MCF-10A cell viability under low attachment conditions caused by adrenoceptor agonists was further investigated by testing their effects in the absence or in the presence of propranolol (10 μM). As shown in Figure 8E, propranolol alone did not alter cell viability under low attachment conditions but prevented the increase in cell viability caused by either isoprenaline or salbutamol (tested at 10 μM).

**Figure 9:**
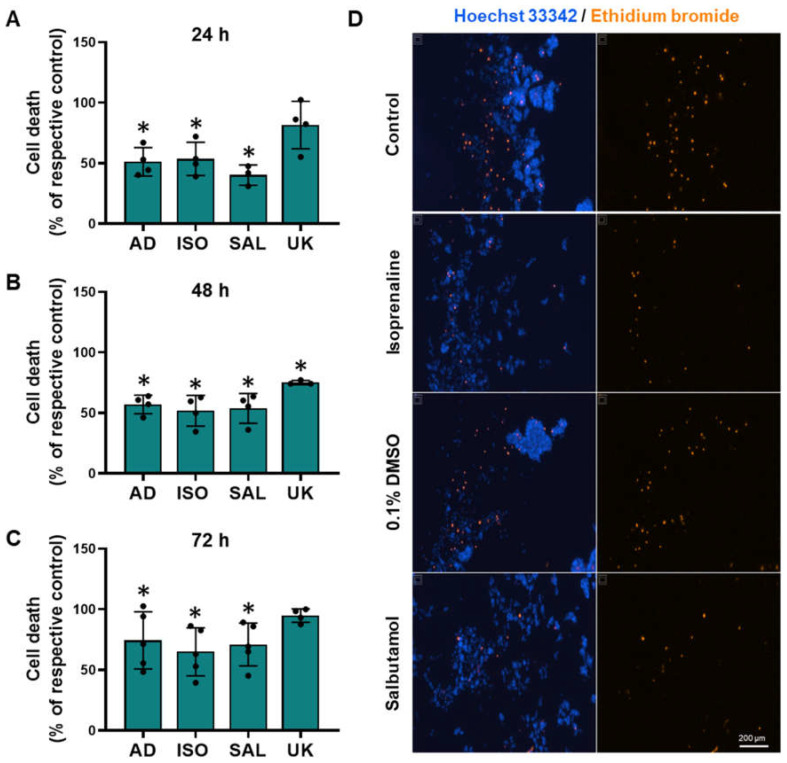
Influence of β- and α_2_-adrenoceptor agonists on MCF-10A cell death under low attachment conditions. (**A**–**D**) Results for MCF-10A cell death under low attachment after treatment with either the non-selective adrenoceptor agonist adrenaline (AD; 10 μM), the non-selective β-adrenoceptor agonist isoprenaline (ISO; 10 μM), the β_2_-adrenoceptor agonist salbutamol (SAL; 10 μM) or with the α_2_-adrenoceptor agonist UK 14,304 (UK; 10 μM) for 24 h (**A**), 48 h (**B**) or 72 h (**C**). Significantly different from solvent: * *p* < 0.05; Student’s *t*-test (**D**) Representative images of ethidium bromide (orange fluorescence) stained MCF-10A cells treated with either 10 μM isoprenaline or 10 μM salbutamol, in parallel with the respective solvent for 24 h. Cell nuclei were stained with Hoechst 33342 (blue fluorescence). Scale bar: 200 μm.

To further provide evidence for the protective effect of adrenoceptor activation on cell survival under low attachment conditions, the MCF-10A cells were incubated with ethidium bromide, which stained dead cells. Treatment with β-adrenoceptors agonists (adrenaline, isoprenaline, and salbutamol, all tested at 10 μM) resulted in a decreased cell death under low attachment conditions (Figure 9). Interestingly, treatment with the α_2_-adrenoceptor agonist UK 14,304 (10 μM) also decreased MCF-10A cell death (Figure 9), although not as effective as the agonists with affinity for β-adrenoceptors. Figure 9D shows representative images of ethidium bromide- and Hoechst 33342-stained MCF-10A cells treated with either isoprenaline or salbutamol (tested at 10 μM), showing a decrease in the number of dead cells.

Cell–cell aggregation is a process that can occur when cells are exposed to low attachment conditions and is believed to be a protective mechanism against cell death under low attachment conditions [60,61]. MCF-10A cells also form cell–cell aggregates with the formation of multicellular structures, when plated under low attachment conditions (see Figure 9D). Treatment with β-adrenoceptors agonists (adrenaline, isoprenaline, and salbutamol, all tested at 10 μM), did not alter cell aggregation, as evidenced by no differences in isolated cells (Supplementary Figure S6). However, β_2_-adrenoceptor activation altered the size distribution of cell aggregates: the MCF-10A treated with β-adrenoceptors distributed mostly in small aggregates, whereas at higher sizes very few aggregates were found, with an approximately 80% reduction compared to control (10 μM adrenaline: 23.4 ± 9; 10 μM isoprenaline 19.3 ± 8; 10 μM salbutamol 11.0 ± 15). The activation of α_2_-adrenoceptors with the selective agonist UK 14,304 did not alter cell aggregation (Supplementary Figure S6). 

## 4. Discussion

Increasing evidence suggested a connection between exposure to stress and an increased risk of developing breast cancer [16,17,18]. The adrenergic system has been implicated in stress-induced cancer development, since adrenaline and noradrenaline are massively released during stress conditions [62] and adrenoceptors activation significantly increase cancer incidence in preclinical animal models [5]. The mechanism by which adrenoceptor activation may induce breast cancer initiation remains poorly understood. The present study reveals that β_2_-adrenoceptor activation on MCF-10A non-tumorigenic breast cells promoted an epithelial phenotype, an increase in cell adhesion, and resistance to cell death, whereas it decreased cell migration, motility, and cell proliferation. Therefore, from a functional perspective, β_2_-adrenoceptors activation can have an important role in breast epithelial cells, which may impact the breast to acquire tumorigenic properties. The hypothesis that β_2_-adrenoceptors are involved is based on indirect pharmacological evidence. We used agonists and antagonists within concentration ranges deemed appropriate for identifying β_2_-adrenoceptor-mediated effects. Therefore, it is reasonable to interpret the observed effects as being primarily mediated by β_2_-adrenoceptors.

In the present study, MCF-10A breast cells were used as a non-tumorigenic cell model. These cells have been reported to be of basal phenotype [63,64] or of a mixture of both luminal and basal cells [65], and have been extensively used by others as a useful model to study early breast carcinogenesis [10,66,67]. The activation of the β-adrenoceptors potentially increased the expression of E-cadherin, an epithelial marker, whereas it decreased the expression of mesenchymal markers, N-cadherin and vimentin. These observations were interpreted as indicating that β-adrenoceptor activation is promoting a mesenchymal–epithelial transition (MET) in MCF-10A cells. MET is the reverse process of epithelial–mesenchymal transition (EMT) through which mesenchymal cells regain epithelial properties [68]. The relationship between MET and carcinogenesis is complex, and its most accepted role is to promote the differentiation of mesenchymal metastatic cells and the formation of secondary tumors [32]. A β-adrenoceptor-mediated induction of MET was previously reported in other non-tumorigenic cells (bronchial epithelial cells [69]) and in cancer cell models, namely in oral squamous cancer cells [33]. A β-adrenoceptor mediated induction of MET may also occur in prostate cancer cells, since β-adrenoceptors inhibition was shown to result in increased mesenchymal markers expression [70]. To our knowledge, the present study is the first to show that, in breast cells, β-adrenoceptor activation can induce MET.

MET has also been associated with alterations in protrusion formation and cell migration [58,59]. The present study corroborates that treatments that promote MET also impair lamellipodia and filopodia formation, in line with the evidence available [71]. Moreover, there is evidence that the activation of β-adrenoceptors can lead to an inhibition of cell protrusions formation (bronchial [72], keratinocytes [73,74], and breast [75] non-tumorigenic cells). In the current study, the impairment of lamellipodia and filopodia formation was observed after treatment with all adrenoceptor agonists tested (isoprenaline, adrenaline and salbutamol) in concentration range with affinity to β-adrenoceptors. Since salbutamol is selective to β_2_-adrenoceptor, the decreased number of cell protrusions are, in all likelihood, mediated by activation of this (β_2_) adrenoceptor subtype.

Protrusions are structures crucial to the motility and migration of the cell [76]. It is widely accepted that treatments that impair formation of cell protrusions impair cell motility [77,78]. The results obtained in the present study are in line with this pattern of effects: β_2_-adrenoceptor activation, which impaired protrusion formation, also reduced single-cell motility and the directional migration of MCF-10A cells. Studies on the role of β-adrenoceptors in cell migration have shown that these receptors can either mediate stimulatory or inhibitory effects on cell migration, depending on the cell type and model used [27,79]. An adrenoceptor-mediated decrease in cell migration was previously shown to occur in non-tumorigenic cells from the bronchi [72], skin [73,74], breast [75], and cornea [80], and in cancer models of the mouth [81], breast [82], prostate [70], and melanoma [79].

Cell migration is controlled by the orchestrated dynamics of cell adhesion and cell protrusions formation [83]. In general, cell motility decreases when protrusion formation is decreased and the effects of cell adhesion proteins are increased [77,78,84,85]. This seems to agree with the results of the present study, which showed that β_2_-adrenoceptor activation increased cell–matrix adhesion in non-tumorigenic breast cells, an observation consistent with previously published results [75,86]. Several cell surface proteins may participate in adhesion [87], and a detailed study of the expression of activity for each protein is methodologically unfeasible. However, it is known that E-cadherin plays a role in the regulation of adhesion, since downregulation of E-cadherin weakens the adhesion of MCF-10A cells to the cell–matrix [88]. In the present study, β-adrenoceptor activation increased E-cadherin levels, indicating that β-adrenoceptor activation favors cell adhesion. The increase in cell adhesion and E-cadherin was associated with cancer cell survival and growth at metastatic niches [32,89,90]. These observations are in alignment with previous research indicating that β-blockers may exert a protective effect on the colonization of various tissues by cancer cells and on relapse-free survival in cancer patients [91,92,93]. Nonetheless, whether the increase in E-cadherin expression in non-tumorigenic breast cells caused by β-adrenoceptor activation may be a contributor to carcinogenesis remains to be proved.

In general, non-tumorigenic cells die under inappropriate attachment conditions [41,42]. In the breast, and from a physiological point of view, the insufficient or inappropriate cell–matrix interactions are important during gland development and homeostasis [45,94]; cells that detach from the gland to the duct lumen will undergo cell death (mainly, from anoikis, that is a particular type of apoptosis) [41,42,95]. Resistance to cell death under conditions where there is inappropriate cell–matrix interactions is a mechanism with pathological relevance, since it can lead to formation of premalignant lesions, such as ductal carcinomas in situ [40,43,44,45]. In the present study, β-adrenoceptor activation reduced cell death under low attachment conditions, which may indicate that after β-adrenoceptor activation, breast cells may be more prone to the development of premalignant lesions/cancer niches. Previous studies have shown that resistance to cell death under low attachment conditions in cervical, ovarian, and liver cancer cells is mediated by β_2_-adrenoceptors [96,97,98]. β_3_-adrenoceptors have also been shown to have protective effects against cell death, namely in melanoma cells [99,100]. In the present study, the profile of the pharmacological responses supports the involvement of β_2_-adrenoceptors. Salbutamol, a selective β_2_-adrenoceptor agonist, attenuated cell death in a similar degree to that is caused by non-selective β-adrenoceptors agonists. Nevertheless, the possibility that α_2_-adrenoceptors may also protect MCF-10A breast cells from cell death must also be considered since UK 14,304, a selective α_2_-adrenoceptor agonist, also attenuated cell death. Taken together, these results suggest that, in breast cells, adrenoceptor-mediated resistance to cell death may be induced by different adrenoceptor subtypes.

It is not known if the type of the cell aggregates is relevant for cell survival, but it is known that cell aggregation is an important mechanism for cell survival under low attachment conditions [60,61,101,102]. In the present study, it was observed that the pattern of aggregation was altered in cells treated with β-adrenoceptor agonists, presenting a more uniform pattern of cell aggregation, predominating small size aggregates. If alterations in the pattern of aggregation reflect changes in the distribution of cell–cell adhesion molecules in the membrane remains to be investigated.

The effects previously discussed indicate that adrenoceptor activation may promote alterations towards the acquisition of a phenotype more favorable to cell survival in the non-tumorigenic breast cells; a pattern compatible with what is expected to observe in tumorigenic cells [103]. However, tumorigenic cells have a higher proliferation rate, normally, which is considered one of the main cancer hallmarks [46]. In the present study, β_2_-adrenoceptors caused a decrease in cell proliferation, showing the inability of the adrenergic stimulation to promote this cancer hallmark. Previously, it has been shown that adrenoceptors activation can decrease cell proliferation in astrocytoma [104], and in breast [105], melanoma [106], and colon cancer cells [107]. In MCF-10A cells, other authors observed identical results in cell proliferation after β-adrenoceptor activation [31,53]. This supports the view that adrenoceptor activation, alone, is unable to promote the acquisition of sustained cell proliferation in these non-tumorigenic breast cells. Therefore, it is conceivable that adrenoceptors may contribute to the acquisition of some hallmarks relevant for tumorigenesis but require other types of stimuli to fully acquire the relevant cancer hallmarks.

In the present experimental conditions, the β_2_-adrenoceptor-mediated effects were observed after a long period of exposure to agonists. Long exposure of β_2_-adrenoceptors to agonists is known to cause a loss of the effects, a process classically called “desensitization” [108]. Recent evidence is showing that desensitization can be seen not as a loss of signaling, but an activation of alternative signaling pathways (non-canonical) capable to control gene expression and cell proliferation [109]. Therefore, the effects described in the present study may reproduce the conditions that may occur in vivo, that promote the transfer of adrenergic signaling from the membrane to a cellular compartment more involved with the control of gene expression.

A persistent adrenergic activation may occur in breast tumors. Recent studies have shown that non-tumorigenic breast cells, when subjected to adrenergic stimulation, begin to express the same enzymes for catecholamine synthesis found in tumorigenic breast cancer cells [51]. This includes tyrosine hydroxylase, the key enzyme in the production of noradrenaline and adrenaline [51]. Consequently, it is conceivable that episodes of stress can provide the stimulus to initiate a tumorigenic transformation and later the adrenaline produced by tumor cells might lead to persistent adrenergic activation within the tumor microenvironment that may favor the spread of tumorigenic properties to adjacent non-tumorigenic cells, promoting tumor growth and development. Appropriate experimental approaches have to be implemented to confirm this hypothesis.

Adrenoceptors are likely to play a pivotal role in the development of a variety of cancers beyond breast cancer. Research indicates that they may be involved in the initiation phase of several cancer types, such as pancreatic cancer [110], lung cancer [111], skin cancer [112], and liver cancer [113]. These findings highlight the need for comprehensive research into adrenergic mechanisms in carcinogenesis, which could significantly inform the potential clinical use of adrenergic antagonists for the prevention and treatment of a wide spectrum of cancers.

## 5. Conclusions 

The present study showed that, in human breast cells, adrenoceptors (mainly β_2_-adrenoceptors) regulate several processes that may promote the acquisition of tumorigenic properties, providing a mechanistic explanation for the increase in breast cancer incidences that may be caused by the adrenergic stimulation known to occur during stress. However, adrenergic stimulation may be seen more as a risk factor than a fully tumorigenic pathway, since not all cancer hallmarks may be induced by adrenoceptor activation in non-tumorigenic breast cells.

## Data Availability

The data presented in this study are available on request from the corresponding author.

## Supplementary Materials

The following supporting information can be downloaded at: https://www.mdpi.com/article/10.3390/cells13030262/s1.
