# Supplementary material for: β2-Adrenoceptor Activation Favor Acquisition of Tumorigenic Properties in Non-Tumorigenic MCF-10A Breast Epithelial Cells
Source: Cells. 2024 Jan 30;13(3):262. doi: 10.3390/cells13030262 (PMC10854540; doi:10.3390/cells13030262)
Supplement: Supplementary file 1 [file cells-13-00262-s001.zip › cells-2770466-supplementary.pptx]

## Slide 1
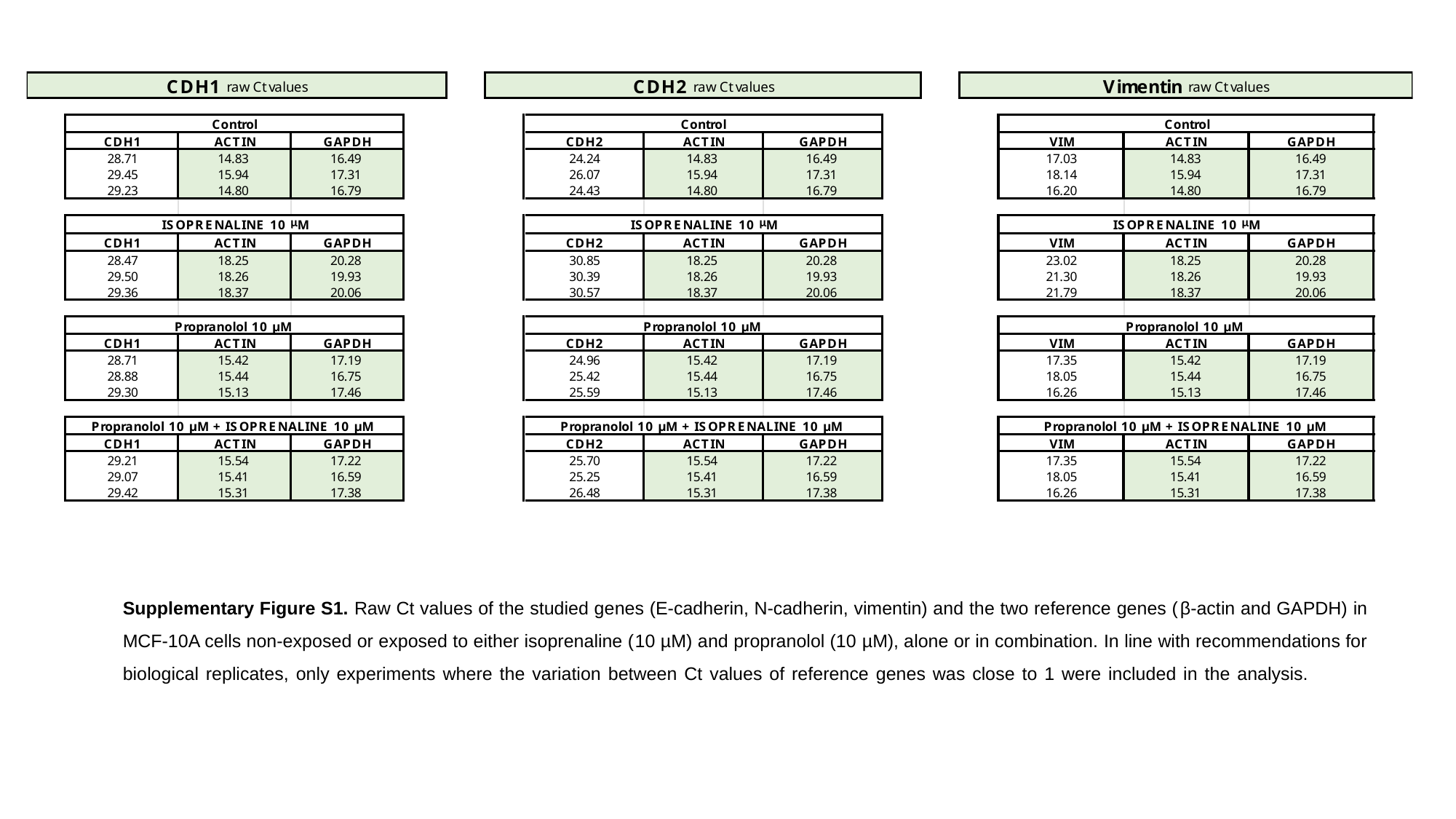

Supplementary Figure S1. Raw Ct values of the studied genes (E-cadherin, N-cadherin, vimentin) and the two reference genes (β-actin and GAPDH) in MCF-10A cells non-exposed or exposed to either isoprenaline (10 µM) and propranolol (10 µM), alone or in combination. In line with recommendations for biological replicates, only experiments where the variation between Ct values of reference genes was close to 1 were included in the analysis.

## Slide 2
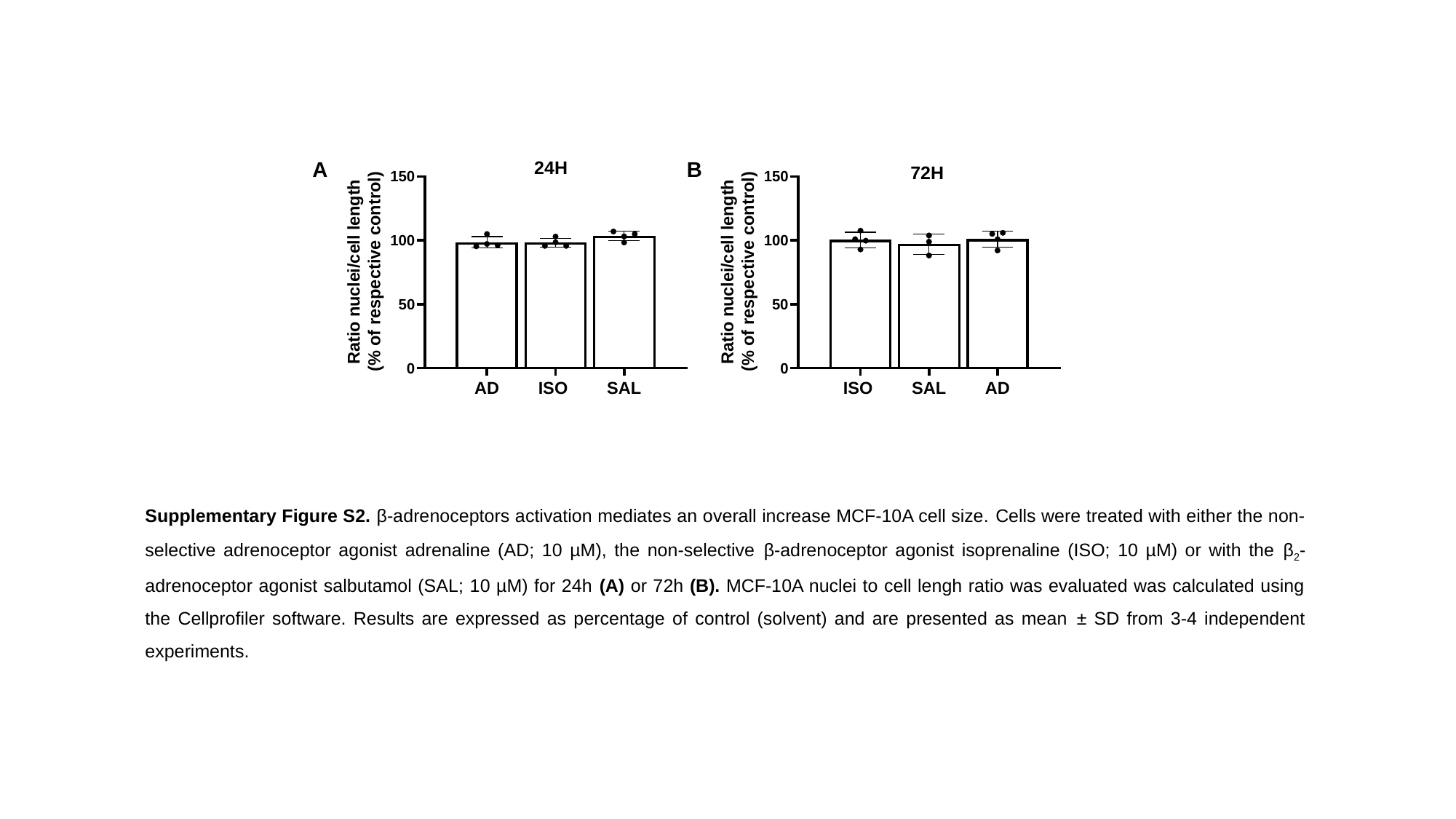

A
24H
B
72H
Supplementary Figure S2. β-adrenoceptors activation mediates an overall increase MCF-10A cell size. Cells were treated with either the non-selective adrenoceptor agonist adrenaline (AD; 10 µM), the non-selective β-adrenoceptor agonist isoprenaline (ISO; 10 µM) or with the β2-adrenoceptor agonist salbutamol (SAL; 10 µM) for 24h (A) or 72h (B). MCF-10A nuclei to cell lengh ratio was evaluated was calculated using the Cellprofiler software. Results are expressed as percentage of control (solvent) and are presented as mean ± SD from 3-4 independent experiments.

## Slide 3
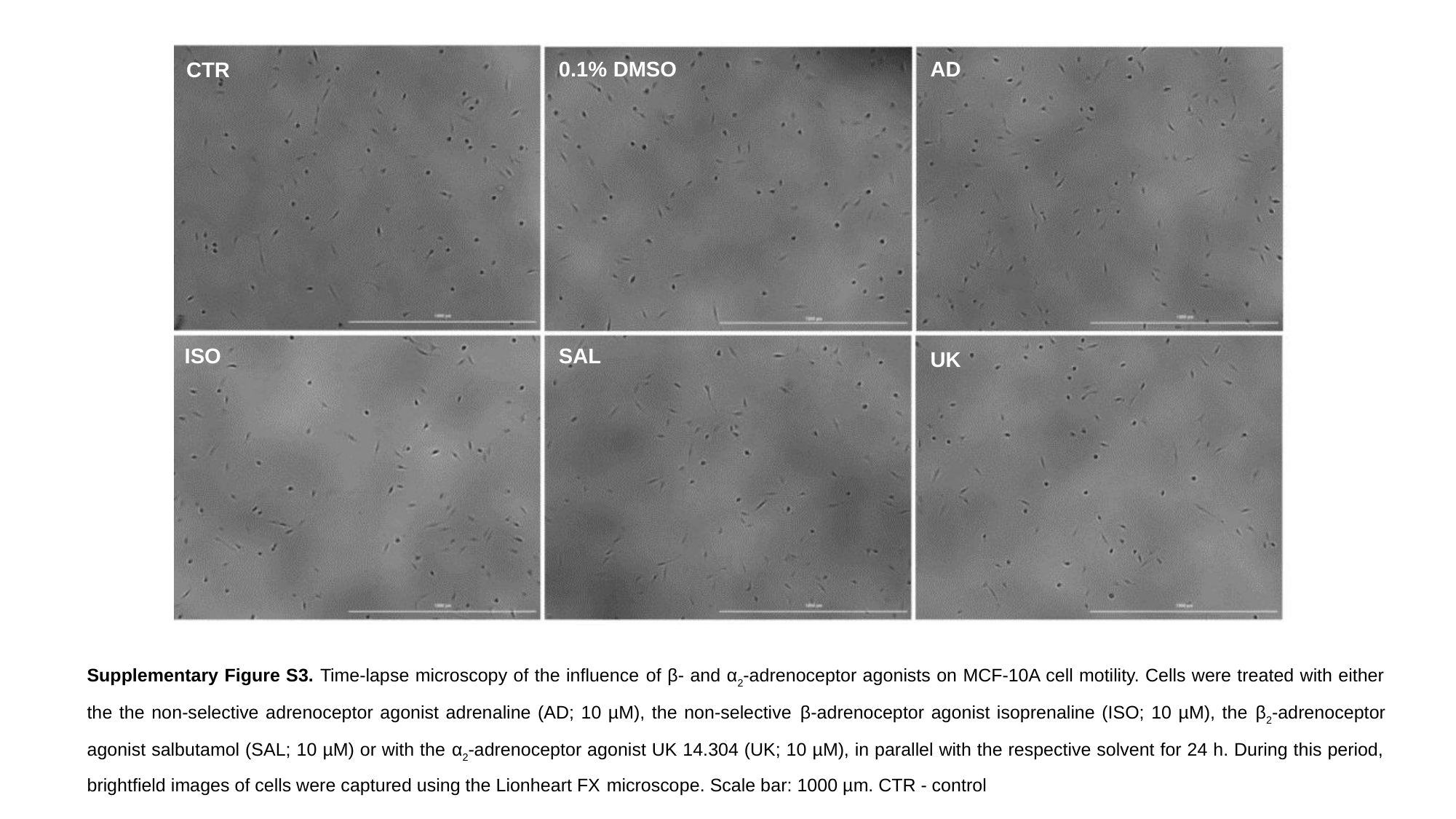

AD
0.1% DMSO
CTR
ISO
SAL
UK
Supplementary Figure S3. Time-lapse microscopy of the influence of β- and α2-adrenoceptor agonists on MCF-10A cell motility. Cells were treated with either the the non-selective adrenoceptor agonist adrenaline (AD; 10 µM), the non-selective β-adrenoceptor agonist isoprenaline (ISO; 10 µM), the β2-adrenoceptor agonist salbutamol (SAL; 10 µM) or with the α2-adrenoceptor agonist UK 14.304 (UK; 10 µM), in parallel with the respective solvent for 24 h. During this period, brightfield images of cells were captured using the Lionheart FX microscope. Scale bar: 1000 µm. CTR - control

## Slide 4
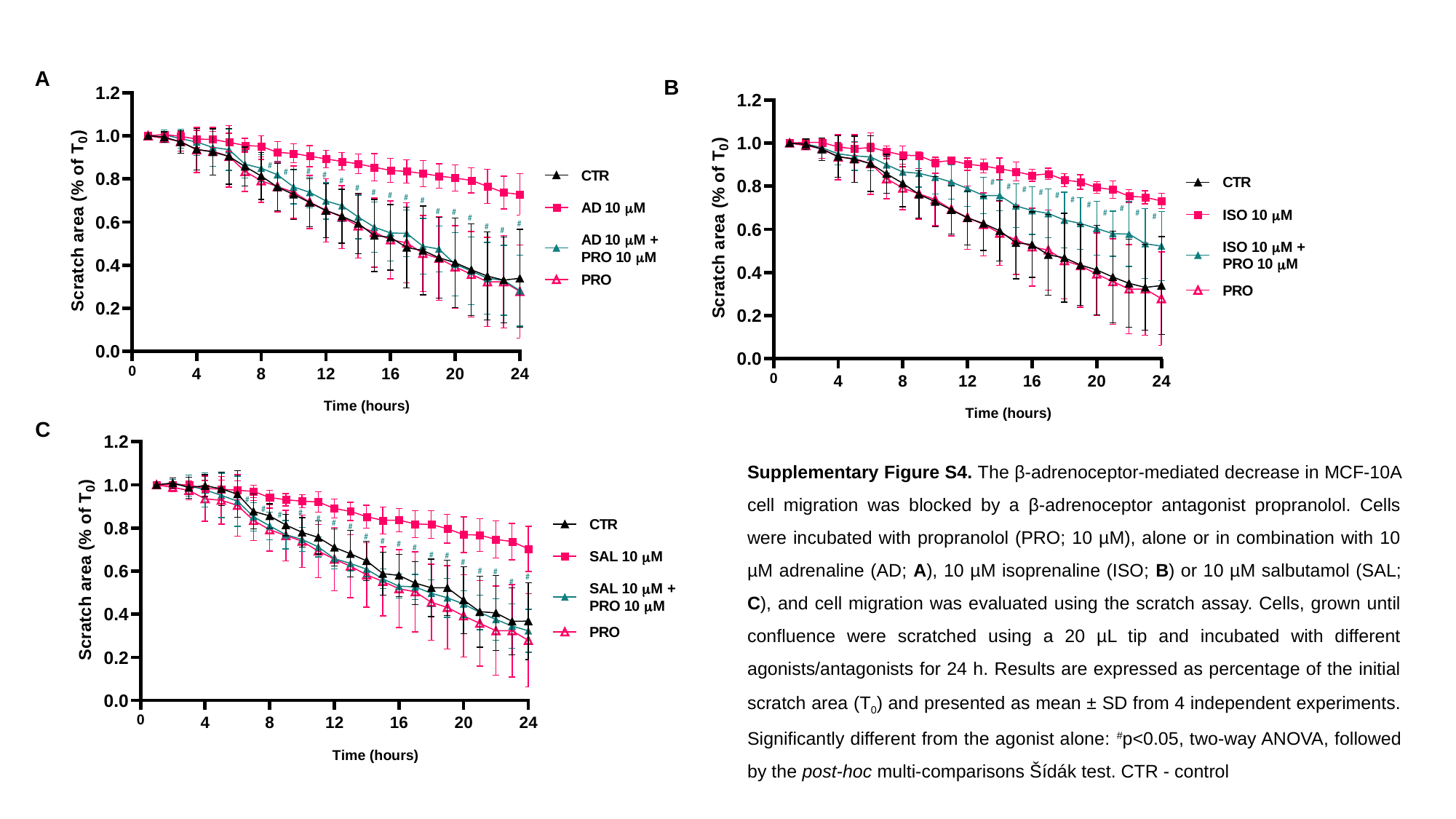

A
B
#
#
#
#
#
#
#
#
#
#
#
#
#
#
#
#
#
#
#
#
#
#
#
#
#
#
#
C
Supplementary Figure S4. The β-adrenoceptor-mediated decrease in MCF-10A cell migration was blocked by a β-adrenoceptor antagonist propranolol. Cells were incubated with propranolol (PRO; 10 µM), alone or in combination with 10 µM adrenaline (AD; A), 10 µM isoprenaline (ISO; B) or 10 µM salbutamol (SAL; C), and cell migration was evaluated using the scratch assay. Cells, grown until confluence were scratched using a 20 µL tip and incubated with different agonists/antagonists for 24 h. Results are expressed as percentage of the initial scratch area (T0) and presented as mean ± SD from 4 independent experiments. Significantly different from the agonist alone: #p<0.05, two-way ANOVA, followed by the post-hoc multi-comparisons Šídák test. CTR - control
#
#
#
#
#
#
#
#
#
#
#
#
#
#
#
#
#
#

## Slide 5
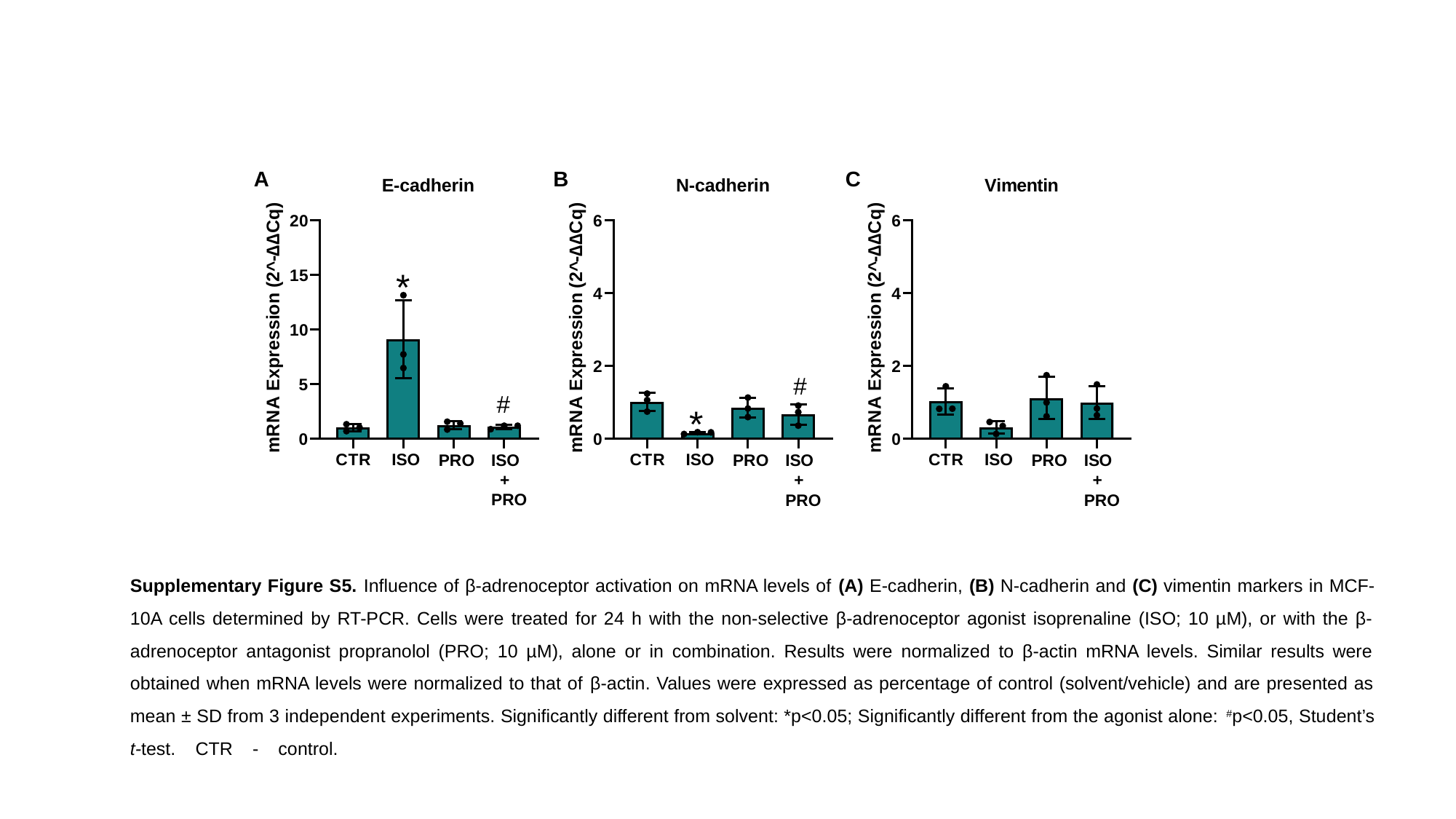

A
B
C
*
#
#
*
Supplementary Figure S5. Influence of β-adrenoceptor activation on mRNA levels of (A) E-cadherin, (B) N-cadherin and (C) vimentin markers in MCF-10A cells determined by RT-PCR. Cells were treated for 24 h with the non-selective β-adrenoceptor agonist isoprenaline (ISO; 10 µM), or with the β-adrenoceptor antagonist propranolol (PRO; 10 µM), alone or in combination. Results were normalized to β-actin mRNA levels. Similar results were obtained when mRNA levels were normalized to that of β-actin. Values were expressed as percentage of control (solvent/vehicle) and are presented as mean ± SD from 3 independent experiments. Significantly different from solvent: *p<0.05; Significantly different from the agonist alone: #p<0.05, Student’s t-test. CTR - control.

## Slide 6
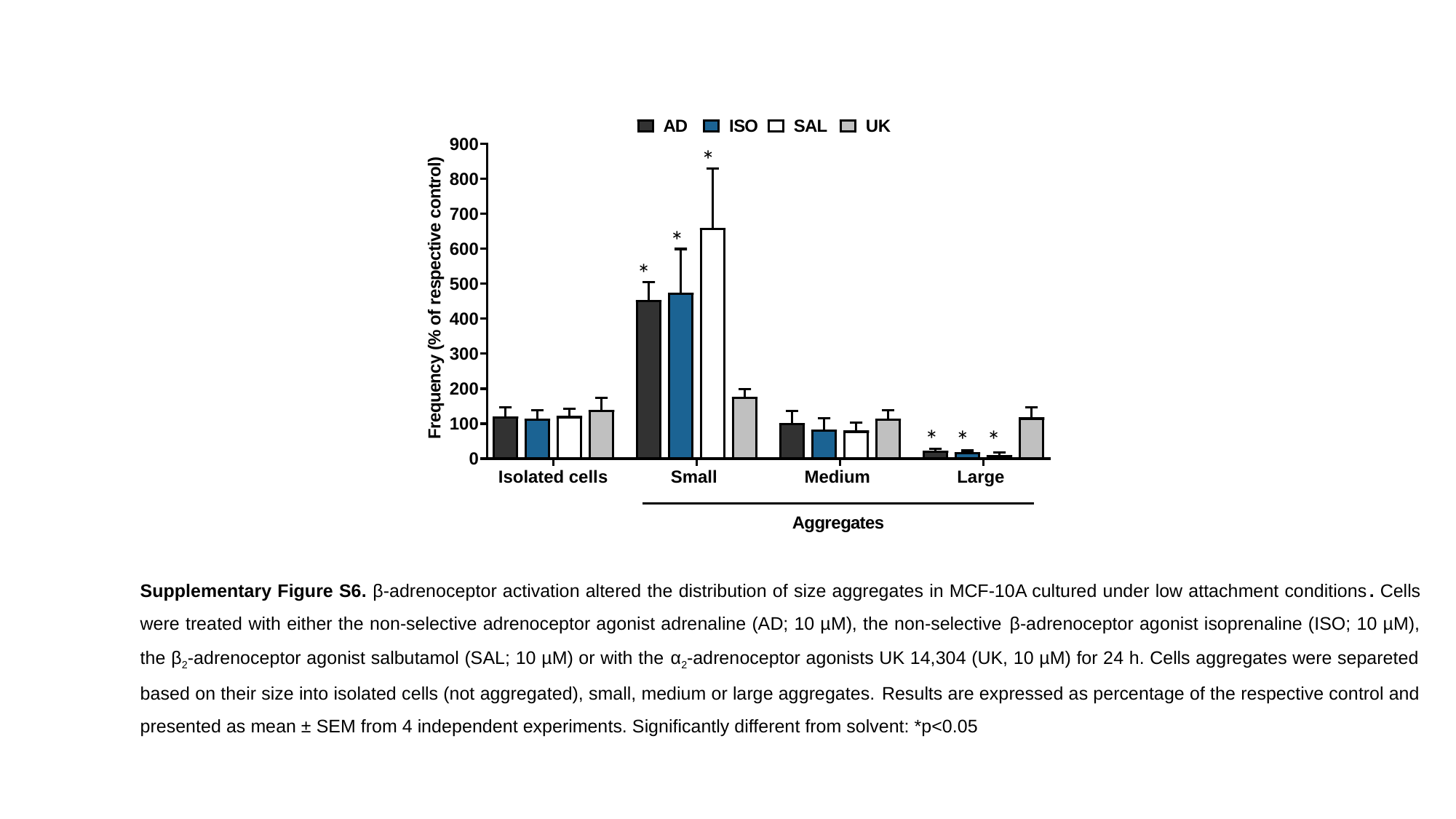

*
*
*
*
*
*
Supplementary Figure S6. β-adrenoceptor activation altered the distribution of size aggregates in MCF-10A cultured under low attachment conditions. Cells were treated with either the non-selective adrenoceptor agonist adrenaline (AD; 10 µM), the non-selective β-adrenoceptor agonist isoprenaline (ISO; 10 µM), the β2-adrenoceptor agonist salbutamol (SAL; 10 µM) or with the α2-adrenoceptor agonists UK 14,304 (UK, 10 µM) for 24 h. Cells aggregates were separeted based on their size into isolated cells (not aggregated), small, medium or large aggregates. Results are expressed as percentage of the respective control and presented as mean ± SEM from 4 independent experiments. Significantly different from solvent: *p<0.05
